# Developing a competency framework for extracorporeal membrane oxygenation nurses: A qualitative study

**DOI:** 10.1002/nop2.1502

**Published:** 2022-12-03

**Authors:** Liwei Hong, Chunyi Hou, Lihua Chen, Xiaoqun Huang, Jingye Huang, Weijuan Liu, Xiangxiang Shen

**Affiliations:** ^1^ Department of Critical Care Medicine Guangzhou Institute of Respiratory Health First Affiliated Hospital of Guangzhou Medical University Guangzhou China; ^2^ Department of Pediatrics First Affiliated Hospital of Guangzhou Medical University Guangzhou China

**Keywords:** competency, critical care nursing, critical incident technique, extracorporeal membrane oxygenation, extracorporeal membrane oxygenation nurse, qualitative research

## Abstract

**Aim:**

To develop a competency framework applicable to Chinese extracorporeal membrane oxygenation (ECMO) nurses.

**Design:**

A qualitative study was performed following the consolidated criteria for reporting qualitative research.

**Methods:**

Semi‐structured interviews based on the critical incident technique were conducted among 21 ECMO care providers recruited from five well‐known ECMO centres in Guangzhou, China. Interview transcripts were coded and analysed using the constant comparative method. The data collection period lasted from November 2021 to April 2022.

**Results:**

A competency framework for ECMO nurses was identified. It included four domains: knowledge, skills, behaviours and attitudes, containing 33 subcompetencies and 66 items.

**Relevance to clinical practice:**

This framework can be a reference for the assessment and training of ECMO nurses.

**What does this article contribute to the wider global clinical community?**

- Based on the critical incident technique, this study developed a competency framework for Chinese ECMO nurses who adopted the single caregiver model.
- The competency framework included four domains: knowledge, skills, behaviours and attitudes, containing 33 subcompetencies and 66 items.
- The framework provides a reference for improving the evaluation criteria and optimizing the training system for ECMO nurses

## 1 INTRODUCTION

Extracorporeal membrane oxygenation (ECMO) has become a new dimension for treating severe cardiorespiratory failure patients currently (Shekar et al., 2014). To date, more than 182,000 ECMO cases have been implemented and 521 ECMO centres have been established globally (Extracorporeal Life Support Organization, 2020). Under the COVID‐19 pandemic, the demand for ECMO treatment has increased significantly (Iwashyna et al., 2020; MacLaren et al., 2020). Multidisciplinary teamwork is required for the successful implementation of ECMO. ECMO nurses play a critical role in multidisciplinary teamwork (Daly et al., 2017). For example, ECMO nurses undertake a variety of significant responsibilities: assisting in establishing ECMO, monitoring the condition of the patient receiving ECMO and the ECMO circuit, transporting, proposing care regimens and implementing the regimen, trouble shooting, providing early rehabilitation for patients and offering follow‐up care after discharge (Alshammari et al., 2022; Lucchini et al., 2019). To provide high‐quality ECMO care, ECMO nurses are expected to possess a high level of competence (Alshammari et al., 2022). However, it has been reported that some competency requirements for ECMO nurses still require to promote, such as leadership and psychological adjustment (Alsalemi et al., 2020; Wellman, 2017).

A synthesis of knowledge, skills, attitudes and values that support the efficient and outstanding performance in a professional or occupational field refers to competency (Dijkstra et al., 2021; Liu & Aungsuroch, 2018). A competency framework refers to a broad range of needed behaviours, serving as a logical framework for admission, development, training and evaluation (Stanford, 2016). In this case, a competency framework for ECMO nurses is quite needed, which can clarify acceptable levels of clinical skills and knowledge and measure competence to identify strengths and weaknesses in ECMO care practice to promote ongoing professional development (Epstein & Hundert, 2002).

## 2 BACKGROUND

A great number of studies have been implemented to emphasize and develop ECMO nurses' competencies. In 2014, to help ECMO nurses be competent in ECMO care, the Extracorporeal Life Support Organization (ELSO) published ELSO Guidelines for training and continuing education of ECMO specialists (ELSO, 2014a, 2014b). Since then, ECMO curricula for local education was composed by ECMO centres referring to the ELSO Red Book (Brogan et al., 2017) and the ELSO Specialist Manual (Brogan et al., 2018). Many individualized training programmes for promoting ECMO nurses' competence were developed based on ELSO guidelines (Assy et al., 2019; Fouilloux et al., 2019; Zakhary et al., 2020). In 2017, an international survey held by Daly indicated that competency training in ECMO care was expected to be included in the ICU nurse training framework further (Daly et al., 2017). In recent years, many studies have been devoted to training and improving basic ECMO cognitive skills (Assy et al., 2019; Fouilloux et al., 2019), although slightly overlooking other core competencies, such as leadership and communication (Alsalemi et al., 2020; Alshammari et al., 2022).

In China, patients receiving ECMO are mainly managed by ECMO nurses, undertaken by ICU nurses. They mainly apply the single caregiver model (Combes et al., 2014). The ratio of patients and nurses is 1:1, which is quite different from other countries (Daly et al., 2017). The nurses were expected to possess great competencies in such a complicated, high‐risk and challenging specialty. Current studies keep a focus on developing competency frameworks for nurses in different areas (Chen et al., 2021; Dijkstra et al., 2021; González et al., 2022; Xu et al., 2022). To our knowledge, generic, particular and national competency frameworks for Chinese ECMO nurses have yet to be established, which limits the group's continuing professional development. The cultivation of competency of ECMO nurses is not only the premise to realize the construction of an ECMO specialist team but also an important base to implement professional care and improve nursing quality for ECMO‐supported patients. The critical incident technique (CIT) is a data collection approach highly recommended for competency identification of different fields (Schluter et al., 2008; Viergever, 2019). The technique includes the STAR principle, which is usually conducted combined with semi‐structured interviews (Schluter et al., 2008). Researchers could use it to collect key information in important events participants faced during working to capture competencies needed to perform efficiently. The Grounded theory research is one of the methods applied in the qualitative study (Turner & Astin, 2021). It originated from the social sciences but has been widespread applied in education and health research (Turner & Astin, 2021). It seeks to generate a theory that is grounded in data and shaped by the views of participants, thereby moving beyond description and towards a theoretical explanation of a process or phenomenon (Corbin & Strauss, 2008). Hence, this study aimed to develop a competency framework for Chinese ECMO nurses through the Grounded theory and the CIT.

## 3 METHODS

### 3.1 Study design

The qualitative study was guided by the Grounded theory approach advocated by Strauss and Corbin (1998). Semi‐structured interviews were delivered based on the STAR principle of the CIT (Schluter et al., 2008; Turner & Astin, 2021). We followed the Consolidated Criteria For Reporting Qualitative Research (COREQ) (Tong et al., 2007) guidelines in reporting the method and the findings (File S1).

### 3.2 Setting

Five adult ICUs of 5 well‐known ECMO centres in Guangdong province, China, were selected. These ECMO centres are ECMO training bases and conducted over 50 ECMO programmes each year. They offer professional education and training for those healthcare providers, who prepare to implement ECMO treatment in the local hospital.

### 3.3 Sampling

Theoretical sampling and purposive sampling were adopted to select participants in this study (Turner & Astin, 2021). First, researchers briefly explained the study to the potential participants, clarified what acquires to be involved, including a commitment to confidentiality and anonymity in publishing the findings, and answered any questions. Second, a consent form, semi‐structured interview guideline and participant information sheet were distributed to participants in advance to obtain adequate preparation.

#### 3.3.1 Recruitment of ECMO specialist nurses

ECMO specialist nurses were eligible to participate if they have worked in ECMO care independently for at least 3 years and delivered care for more than 30 patients receiving ECMO each year in the last 3 years.

#### 3.3.2 Recruitment of ECMO physicians

Since ECMO physicians witnessed the relative nursing competencies during multidisciplinary teamwork, we included ECMO physicians as a group to gain a valuable perspective. Physicians were eligible to participate if they have worked in ECMO care independently for at least 3 years and implemented ECMO programmes for more than 30 patients each year in the last 3 years.

#### 3.3.3 Recruitment of ECMO centre managers

ECMO centre managers were eligible to participate if they were the head of the critical care department and directed implementing ECMO programmes for over 5 years.

#### 3.3.4 Recruitment of ECMO educators

ECMO educators were eligible to participate if they were in a senior title and supervisors of ECMO nurses for more than 10 years.

### 3.4 Data collection

The data collection period lasted from November 2021–April 2022. Semi‐structured interview guides were used in all interviews. The guide for each group is not quite the same (File S2). The guides were developed based on the STAR principle of the CIT and literature review relating to nurses' roles, responsibilities and competencies in ECMO care (Alshammari et al., 2022; Daly et al., 2017; Melnikov et al., 2021). Two ECMO specialist nurses who met the participant selection criteria were invited to pre‐test the guide. The first and corresponding authors examined and refined the questions and arranged them into a logical flow from wide to specific focus. During the course of the interview study, several questions were revised (e.g. the phrasing was simplified) or supplemented to clarify important content arising in the earlier interviews (Nordfonn et al., 2019).

All interviews were conducted face‐to‐face and audio‐recorded. Two researchers (X1, X2) are graduate students who systematically studied the relevant methodology of qualitative research before and conducted the interview. X1 was responsible for leading the interview. X2 was responsible for observing and recording participants' non‐verbal messages, such as facial expressions and movements. The researchers introduced themselves at the commencement of the interviews to create rapport with each other. Only participants were allowed to attend the interview session to ensure privacy. Each participant was interviewed once. After ending the interview, the first author transcribed interviews verbatim in 24 h. The co‐authors checked the transcripts for accuracy. Field notes were taken throughout the data collection process to capture any key insights arising from the interview. Each participant's transcript was clearly marked and saved separately in a MS Office file.

### 3.5 Data analysis

Data analysis was guided by the coding procedures and constant comparative method of Strauss and Corbin (1998). The analysis process began with a detailed reading of the raw text to become familiarized and made a good understanding of the content. We mainly followed the following steps (Foley & Timonen, 2015). First, focusing on identifying ECMO nurses’ competencies to promote the nursing quality and guarantee the safety of ECMO care, the transcripts were divided into analytical units, defined as meaningful sentences. Two of the authors (X1 and X2) coded independently. Notes of thoughts and memos were recorded throughout the analysis process. Second, open coding was applied to extract codes. The data were organized into competencies requirements, and similarities and differences in the semantic content of the codes were used to generate concepts in subcompetencies. Then, by exploring tentative relationships between subcompetencies and domains, subcompetencies described domains in more detail. Next, the abstraction level was increased to form domains using the associations among subcompetencies. Associations between domains were investigated and structured to form a competence framework. The coders compared, discussed and resolved the differences, and the other co‐authors joined in if required. The interview study stopped when no new code was added to the framework, and when the study's aim had been achieved. NVivo software version 11.0 was used for processing and comparing the codes.

### 3.6 Trustworthiness

Trustworthiness in the qualitative study consists of credibility, confirmability, transferability and dependability (Hewitt et al., 2014). It is guaranteed by the methodology and design of the study. In this study, credibility was ensured by the participants. The participants recruited in this study represented different professional groups involved in ECMO care, and were able to ensure that the developed competency framework was comprehensive and specific to contemporary ECMO nursing practice. Besides, confirmability was demonstrated as all data were collected from identifiable sources in this study (Zhang et al., 2019). The aim to develop a competency framework suitable for Chinese ECMO nurses confirmed transferability. The competency framework constructed is thus generalizable to ECMO nursing settings throughout China and other countries which serve the same caregiver model. The dependability of findings was indicated if results can be repeated by other scholars (Hewitt et al., 2014). In this study, three more ECMO care managers were invited to review the final competency framework to guarantee the accuracy of the findings. Undoubtedly, as ECMO nursing develops, it will be necessary to conduct research using the same design. In addition, as the participants change, participants' interests and ideas will shift, as will the results of future studies. Therefore, the findings of this study are reliable.

## 4 RESULTS

### 4.1 Demographic characteristics

From the five centres, 21 workers participated in the interviews. The detailed characteristics of the study participants are presented in Table 1. They were ECMO specialist nurses (*n* = 12), ECMO physicians (*n* = 2), department head nurses (*n* = 6) and educator (*n* = 1). Most participants were female (14/21). The mean (SD) year of experience in ECMO care is 7.4 (3.5). The mean length of interviews was 28.5 min (13–60 min). Participants mentioned 40 episodes total, covering complication management, emergency response, ECMO teaching, humanistic care and communication with the patient's family.

**TABLE 1:** Participants' demographic characteristics

Characteristics	Frequency	Percent (%)
Age range		
30–40	14	66.7
41–50	6	28.6
51–60	1	4.8
Gender		
Female	14	66.7
Male	7	33.4
Highest level of education		
Bachelor's degree	19	90.5
Master's degree	2	9.5
Tittle		
Intermediate	16	76.2
Senior	5	23.8
Year of experience in critical care		
5–10	6	28.6
11–15	6	28.6
16–20	6	28.6
21–25	2	9.5
>25	1	4.8
Year of experience in ECMO care		
3–5	8	38.1
6–10	10	47.6
>10	3	14.3
Role		
Manager	6	28.6
Educator	1	4.8
ECMO physician	2	9.5
ECMO nurse	12	57.1
Number of cases discussed in the interviews		
Successes	26	65.0
Failures	14	35.0
Case type		
Complication management	3	7.5
Emergency response	33	82.5
ECMO teaching	1	2.5
Humanistic care	2	5.0
Communication with the patient's family	1	2.5

### 4.2 Competency framework for ECMO nurses

**FIGURE 1:**
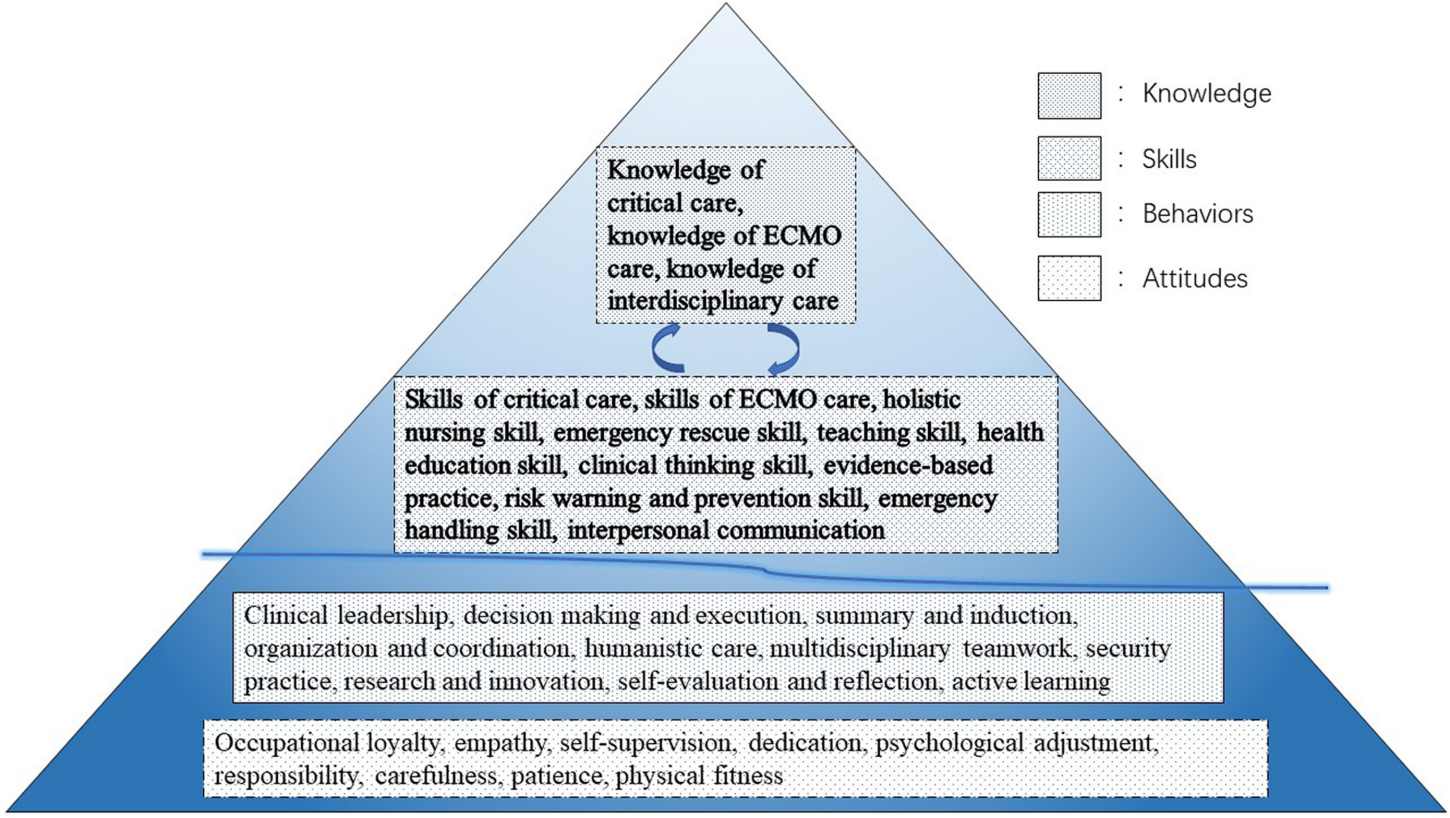
Competency model for extracorporeal membrane oxygenation nurses in China, based on the Iceberg Model. This shows the structure of extracorporeal membrane oxygenation nursing competencies required in the model of China. The surface layers of the Iceberg Model contain three subcompetencies of knowledge and 11 subcompetencies of skills. The deeper layers contain 10 subcompetencies of behaviours and nine subcompetencies of attitudes.

A competency framework for ECMO nurses was identified, which includes four domains, encompassing 33 subcompetencies and 66 items. The four domains were “knowledge;” “skills;” “behaviours;” and “attitudes.” The details of the competency framework are shown in Tables 2, 3, 4, 5. To visualize the framework, the domains and subcompetencies were structured using the Iceberg Model (Spencer & Spencer, 1993). These are shown in Figure 1 as a competency model for ECMO nurses. The domains generated in this study are described specifically below with supporting verbatim phases.

**TABLE 2:** Knowledge of ECMO nurses

Domains	Subcompetencies	Items in subcompetencies
Knowledge	Knowledge of critical care	Familiarizing with the basic medical knowledge of critical care, such as the anatomy, physiology and pathology of the circulatory system, respiratory system, urinary system, digestive system and so on; familiarizing with the pathogenesis and observation of different primary diseases and other relevant knowledge.
		Mastering knowledge of critical care nursing, such as fluid management strategies, using approaches of common assessment tools such as ICU sedation score, analgesia score, organ failure score and Acute Physiology and Chronic Health Evaluation II (APACHE II), and preventive measures for deep vein thrombosis.
		Familiarizing with the interpretation methods of clinical laboratory examination indicators, the mechanism of action and using methods of common rescue drugs, and emergency rescue procedures, such as cardiopulmonary resuscitation (CPR).
		Mastering the professional knowledge of principles of extracorporeal circulation and advanced life support techniques commonly used in critically ill patients, such as prone positioning (PP), continuous renal replacement therapy (CRRT), mechanical ventilation, intra‐aortic balloon pump (IABP) and pulse indicator continuous cardiac output (PiCCO).
	Knowledge of ECMO care	Familiarizing with the principles, indications, contraindications, the timing of initiation, physiological characteristics, principles of setting each parameter, required conditions for puncturing vessels, required medical environment and performances of ECMO equipment and relevant consumables for treating critically ill patients in two different modes, venovenous ECMO and venoarterial ECMO.
		Mastering the procedures and key points of nursing cooperation of ECMO pipe pre‐flush, catheterization, pipe fixation, pipe maintenance, membrane replacement and programmed weaning.
		Grasping the key points of observations during the application and after weaning of different ECMO modes.
		Mastering the key points of monitoring the four pipes of the ECMO equipment during the operation, including waterway, circuit, air route and oxygenator access.
		Familiarizing with theoretical knowledge related to physiological and pathological changes in the patient's respiratory mechanics, circulatory system, digestive system, immune system and coagulation mechanism during ECMO treatment and the target levels and rationale of relevant laboratory indicators.
		Familiarizing with the clinical manifestations, key points of recognition, principles of management and preventing measures of common ECMO‐associated complications, such as bleeding, thrombosis, nosocomial infection, air embolus, lower limb ischemia and so on.
		Familiarizing with relevant knowledge about the timing of ECMO weaning, management strategies for programmed weaning and key points of long‐term follow‐up.
		Understanding the timing of initiating “awake” ECMO and grasping the key points of nursing for patients with “awake”ECMO.
		Mastering the nursing key points for long‐term ECMO.
	Knowledge of interdisciplinary care	Familiarizing with knowledge about infection control, rehabilitation nursing, psychology, pharmacology, occupational safety and self‐protection during ECMO care.

**TABLE 3:** Skills of ECMO nurses

Domains	Subcompetencies	Items in subcompetencies
Skills	Skills of critical care	Being able to comprehensively evaluate the physiological and psychological conditions in critically ill patients to complete the taking of medical histories and systematically and comprehensively observe their conditions according to changes in the respiratory system, circulatory system, digestive system, etc., and carry out critical care assignments in an organized manner.
		Being able to correctly and effectively carry out all critical care techniques, including basic nursing techniques such as oral nursing and nasal feeding and specialized nursing techniques such as closed suction and deep vein indwelling and nursing, and master the implementation and nursing essentials of usual basic and advanced life support techniques such as CPR, PP, CRRT, IABP and mechanical ventilation.
		Being able to accurately implement liquid management strategies.
		Being able to collect test specimens correctly and normatively.
		Being able to write medical documents accurately, timely and truthfully.
	Skills of ECMO care	Completing the preparation independently, quickly and accurately before establishing ECMO, including materials preparation, patient preparation, environment preparation and team preparation.
		Completing ECMO pipes pre‐flush independently, accurately and quickly.
		Completing nursing cooperation of ECMO establishment, pipe fixation, pipe maintenance, membrane replacement and programmed weaning procedures under different modes independently.
		Being able to check the safety of ECMO patients and ECMO circuit accurately, including checking the connection and fixation status of each pipe and parameter values and pressure monitoring of pre‐membrane and postmembrane timely.
		Being able to effectively observe patients' vital signs, the dryness of the puncture site dressing, the recovery level of each system, the operation status of each ECMO pipe and the effectiveness of the ECMO membrane during treatment with different ECMO modes.
		Being able to use various assessment tools for critically ill patients correctly, such as the ability to assess patients' level of sedation and analgesia and then properly restrain ECMO patients.
		Being able to evaluate and monitor the cardiopulmonary function of ECMO patients correctly, use anticoagulant drugs and implement anticoagulant monitoring and management correctly, and reasonably interpret the results of usual laboratory tests such as blood gas analysis, infection indicators and coagulation indicators.
		Being able to formulate individualized rehabilitation training programmes covering the early, middle and long‐term stages of ECMO treatment for ECMO patients according to their conditions and assist them in carrying out progressive rehabilitation training in various forms, such as active training and passive training.
		Mastering transport procedures of ECMO patients and completing the inter and intra‐hospital transport quickly, safely and effectively.
		Being able to take corresponding nursing measures to prevent and treat ECMO‐associated complications and strengthen infection prevention and control, anticoagulant and haemorrhage monitoring, and ventilator‐associated pneumonia prevention and management for long‐term ECMO.
		Being able to implement aseptic techniques and other infection control relevant preventive measures strictly during delivering ECMO care.
		Being able to make plans for long‐term follow‐up and implement them.
		Being able to charge correctly according to ECMO‐related charging standards.
	Holistic nursing skill	Being able to view critically ill patients and applications of ECMO as a complex holistic, provide care effectively for patients treated with both ECMO and other conventional therapeutic techniques and instruments such as PP, CRRT, IABP and mechanical ventilation.
	Emergency rescue skill	Mastering first aid techniques and being able to quickly and calmly implement the necessary emergency rescue measures, such as CPR, when faced with an emergency ECMO patient treatment scenario.
	Teaching skill	Being able to develop systematic teaching plans, carry out various ECMO‐related training activities such as lectures and workshops for low‐level nurses, complete the teaching and assessment tasks for new trainees, and guide and assist low‐level nurses on ECMO care to help cultivate ECMO nursing talents and improve the overall quality of the ECMO team.
	Health education skill	Providing health education and counselling to ECMO patients and their family members based on principles and methods of health education.
Skills
	Clinical thinking skill	Being able to apply knowledge such as nursing, anatomy, pathology, physiology, pharmacology and psychology making a health assessment to analysis, analogy, integrate, conjecture, evidence‐based confirm or exclude, inductive, and then develop individualized care plans and implement them critically, and constantly revise the thought process and thought activity.
	Evidence‐based practice	Being able to integrate evidence from domestic and foreign advanced research such as the latest guidelines and guide ECMO nursing practice based on evidence‐based concepts to explore suitable nursing methods for national conditions.
	Risk warning and prevention skill	Being able to evaluate predictively and early warning about the potential risk factors that may induce ECMO‐associated complications such as coagulation dysfunction, haemorrhage, thrombosis, lower limb ischemia, infection, air embolism and adverse events like inadvertent decannulation and pressure ulcers, and implement corresponding nursing interventions to prevent the occurrence of accidents.
	Emergency handling skill	Mastering relevant ECMO emergency plans such as circuit disruption, inadvertent decannulation, raceway rupture, system or component alarm elimination, air embolus, intracranial and other haemorrhage and so on.
		Being able to respond quickly and flexibly in the above emergencies.
Interpersonal communication	Communicate with ECMO multidisciplinary teams, patients and family members timely, accurately and effectively.

**TABLE 4:** Behaviours of ECMO nurses

Domains	Subcompetencies	Items in subcompetencies
Behaviours	Clinical leadership	Training ECMO technical teammates in echelon style, being able to analyse colleagues' capacity comprehensively to schedule and delegate ECMO care tasks reasonably.
		Controlling and managing the quality performed by low‐level nurses timely, feedbacking timely.
		Leading building ECMO teams, writing ECMO‐related nursing procedures and guidelines, and assisting the head nurse in managing and promoting the development of the ECMO team.
	Decision‐making and execution	About the patient as the focus in the process of ECMO care and making reasonable decisions quickly; taking action quickly without hesitation and completing their work plan or target on schedule with high quality.
	Summary and induction	Accumulating, comparing and analysing the experience of ECMO care regularly, summarizing the nursing points of special cases and rare cases to provide guidance and reference for clinical practice.
	Organization and coordination	Coordinating the own working priorities reasonably.
		Organize colleagues to assist the team in completing ECMO treatment.
		Carrying out effective coordination with colleagues and leaders in different departments for ECMO support patients.
	Humanistic care	Providing relevant humanistic care, especially for “awake” ECMO patients, offering understanding and encouragement to relieve their negative emotions to improve patients' cooperation and compliance, providing psychological care for their family members, and assisting in establishing stress‐facing systems.
	Multidisciplinary teamwork	Obeying the arrangement and willing to cooperate with others in the multidisciplinary ECMO team to complete implementing ECMO programmes.
	Safe practice	Keeping awareness of legal and carrying out ECMO nursing work strictly following nursing standards to keep themselves and patients safe.
	Research and innovation	Possessing scientific literacy, being able to use nursing research and other related scientific research methodologies conducting literature retrieval, carrying out ECMO nursing research, and writing research articles.
		Being able to put forward relevant insights and innovative ideas in ECMO care, such as puncture methods, pipe fixation methods, dressing replacement methods, pain‐reducing methods, skin pressure ulcer prevention and treatment and so on, and try ECMO nursing practice creatively.
	Self‐evaluation and reflection	Conducting self‐reflection regularly, evaluating objectively, clarifying strengths and weaknesses in ECMO care and being able to seek help in time.
	Active learning	Keeping learning‐relevant ECMO knowledge and strengthening ECMO nursing skills through online learning and attending conferences; willing to delve into and explore related frontiers of ECMO disciplines, including new knowledge, new technologies and new ideas.

**TABLE 5:** Attitudes of ECMO nurses

Domains	Subcompetencies	Items in subcompetencies
Attitudes	Occupational loyalty	Owning a correct career outlook, recognizing and evaluating the professional status and responsibility of critical care nursing positively, loving ECMO nursing career, acknowledging career planning, so as to form the cognition and psychological state of adhering to the interest and desire of career choice, career motivation and career loyalty.
	Empathy	Empathizing with the emotions of ECMO patients and their families, understanding and sympathizing with their demands and then committing to a series of activities and processes to improve the outcomes of ECMO patients.
	Self‐supervision	Paying attention to the own behaviours and keeping strict self‐supervise in the process of ECMO nursing.
	Dedication	Having dedication, dare to undertake the task of ECMO nursing.
	Psychological adjustment	Being able to resist stress and make psychological adjustments timely, adapt to the high risk and challenging environment in ECMO nursing quickly and complete the ECMO care work successfully.
	Responsibility	Maintaining a strong sense of responsibility in ECMO care.
	Carefulness	Paying attention to the details and situations in the whole process of ECMO care.
	Patience	Treating patients and their families with great patience.
	Physical fitness	Possessing a strong body and fulfilled with energy to meet the comprehensive physical fitness requirements needed in ECMO care.

#### 4.2.1 “Knowledge”‐related competencies

ECMO nurses are advanced ICU nurses who have received systematic and specific ECMO‐related training in the management of the ECMO patient and the ECMO circuit. They must have sufficient knowledge‐related competencies to help implement ECMO treatment and deliver bedside care. The domain of knowledge encompasses three subcompetencies. The details are shown in Table 2.

Most participants emphasized knowledge of critical care is the solid foundation for ECMO care. Familiarizing with the basic medical knowledge of critical care helps deliver care safely.

For some patients on venoarterial ECMO, their hearts were not functioning well. Your massive infusion will induce heart failure. We need to manage these patients from anatomical and physiological aspects. (Participant 9, department head nurse)

Familiarizing with the interpretation methods of clinical laboratory examination indicators helps manage patients more strategically.

We need to pay attention to the ECMO patient's laboratory test results, such as infection indicators and coagulation indicators. In this way, we can predict the changing trend of their conditions in the process of care and take some measures timely. (Participant 18, ECMO specialist)

Mastering knowledge of critical care nursing helps complete tasks on ECMO care successfully.

You need to ensure the patient's sedation and analgesia status is ideal, there is no potential for inadvertent decannulation, the quality of ECMO pre‐flush is compliant, and fluid management strategies are appropriate. You may require mastery of such a series of knowledge. (Participant 12, department head nurse)

Sometimes ECMO was implemented combined with other techniques, such as intra‐aortic balloon pump (IABP) and continuous renal replacement therapy (CRRT). Hence, you need to master the rationale and mechanism when used in combination. (Participant 12, department head nurse)

Besides, knowledge of ECMO care was perceived as the prerequisite for implementing ECMO care.

The most basic knowledge base is needed, right? It includes the whole process of pipe placement, the principle of ECMO, the precautions for ECMO puncture, and the contraindications for ECMO puncture. Although it is up to the physician to judge whether a patient requires ECMO treatment, nurses also need to understand it for working more effectively. For example, the nurse can add and make a reminder by the time any detail is uncertain or missed. (Participant 14, ECMO physician)

More profound is the change of their respiratory mechanics, the effect on the circulatory system, and fluid management after undergoing ECMO, as these will affect their perfusion. (Participant 18, ECMO specialist)

Familiarizing with clinical manifestations, recognition points, management principles and preventive measures of common complications related to ECMO was highly emphasized.

ECMO‐associated complications are very usual. You could see bloody secretions, hematuria, and skin petechiae. Besides, venous thrombosis could also form since staying in bed always. Another is nosocomial infection. If the connector was contaminated, a bloodstream infection may occur and impact the recovery. (Participant 8, ECMO specialist)

To ensure high‐quality care, ECMO nurses were expected to master knowledge of interdisciplinary care.

So which direction will nurses go in the future? It's possible that nurses will play an important role in the rehabilitation of ECMO patients because of human resource issues or cost issues. ECMO nurses are more willing to do it, since physicians feel they have something more urgent to do. (Participant 4, department head nurse)

#### 4.2.2 “Skills”‐related competencies

Participants stated skills play a significant role in ECMO care. To achieve better outcomes for ECMO patients, ECMO nurses were deemed to require mastery of a series of professional skills. The domain of skills encompasses 11 subcompetencies. The details are shown in Table 3.

ECMO patients are also critically ill patients. Long‐term sedation and analgesia can inhibit the cough reflex of ECMO patients, and slow down the bowel movement, as a result, they may get gastric retention and may need to retain a jejunal nutrition tube. Besides, prolonged bed rest is the potential to increase the occurrence of the corresponding complications, like ventilator‐associated pneumonia, and pressure sores. ECMO nurses require to take corresponding measures for them. (Participant 5, ECMO specialist)

For nutrition, the nurse could insert a jejunal nutrition tube without any machine, a skill that requires training. Then according to the patient's tolerance to nutrition solutions with different osmotic pressure, we selected the appropriate nutrition, set nutrition targets, and adjusted the rate of infusion. We also try to correct the hypoproteinemia and electrolyte disturbances and control the blood glucose levels in patients with combined diabetes. (Participant 2, ECMO specialist)

Foam excipients are used between the ECMO line and the skin. Pillows are padded along the lower leg to elevate the heel. An airbed is used to protect the skin throughout the body and we help change the patients' position once each 2h. We aim to reduce pressure on the skin of the lower limbs, prevent deep vein thrombosis and avoid pressure injuries. (Participant 2, ECMO specialist)

ECMO treatment is risky and families are generally concerned. We will introduce the effects of ECMO treatment before starting. We also communicate with them regularly about their condition and understand their financial status and sources of stress. Some patients who obtained recovery end up not having enough money to pay for it and we will pay for it. (Participant 15, department head nurse)

It seemed difficult to undertake the responsibility unless mastering ECMO care skills, which also was treated as the threshold for ECMO nursing.

One should be familiar with these (ECMO care) skills prior to taking care of the patient, such as completing the preparation independently, quickly, and accurately before establishing ECMO, completing nursing cooperation of ECMO establishment, pipe fixation, pipe maintenance, membrane replacement, and programmed weaning of different models independently. We could supply the theoretical knowledge of ECMO care later. (Participant 21, educator)

ECMO nurses are perceived as mastering the first aid skills and being able to perform quickly and calmly when faced with an emergency scenario.

We just clipped the tube and stopped the pump first. Then someone performed CPR, one called the doctor for resuscitation, and another one prepared the resuscitation materials. 137 pieces of epinephrine were used in such an urgent moment. (Participant 4, ECMO specialist)

Teaching competence is also needed.

Nowadays, when developing an ECMO nurse, it is mainly about the senior nurse mentoring and collaborating with the junior nurse. (Participant 7, ECMO specialist)

ECMO nurses are also supposed to acquire health education skills.

You need to assess patients' compliance and educate them about the purpose of the current use of the ECMO and the consequences of inadvertent decannulation caused by the patient. These should be well educated, especially for ‘awake’ ECMO patients. (Participant 8, ECMO specialist)

Especially, ECMO nurses need to compare the conditions of different patients and make clinical thinking and decision towards different individuals during ECMO care.

For example, for an ‘awake’ ECMO patient, you need to fully assess his compliance, and if he is irritable you need to confirm that restraint is ideal and think about the demand for adding sedative medication because the result of inadvertent decannulation of ECMO pipes can be fatal. (Participant 6, ECMO specialist)

Evidence‐based practice skill is great needed.

Hopefully, our current care measures and decisions can be supported by evidence and convince others. It may be necessary to extract some evidence from foreign countries which have carried out quantities of ECMO programs to explore Chinese ECMO care methods. (Participant 2, ECMO specialist)

Participants all highly emphasized the significance of risk warning and prevention skills.

Being able to detect potential risks in advance is really important. For instance, ECMO nurses should be cautious about risks that whether the patient's condition will cause him to fall out of the pipe, and whether the ECMO keeps efficient or has formed a clot. If you do not discover these conditions in time, all of these could lead to serious consequences. (Participant 1, ECMO specialist)

Among the 40 episodes, 33 emphasized the emergency handling skill.

They must respond to emergencies first and quickly. Otherwise, the patient will miss the opportunity to be treated by the time you call a physician and cannot live due to being completely dependent on ECMO. (Participant 13, ECMO physician)

I was impressed by a previous patient who was on venovenous ECMO and was in a state of reducing sedation dose. He was irritable and ripped out one ECMO pipe. The blood pressure immediately went down. The nurse in charge reacted quickly and clamped the pipe at once. Then he called colleagues. Fortunately, physicians were nearby and begun to perform CPR. At the same time, the patient was quickly provided with blood product infusion and other rehydration. Soon, he was resuscitated and successfully got discharged finally. (Participant 14, ECMO physician)

#### 4.2.3 “Behaviours”‐related competencies

These underpinned 10 subcompetencies of “behaviours.” The details are shown in Table 4. Participants stated the behaviours presented contribute to the excellent nursing teamwork and help improve the comprehensive treatment level of ECMO centres.

Clinical leadership was needed, especially.

These are what trainees need to prepare for before conducting this technology and carrying out an echelon‐type training of ECMO care talents. In addition to mastering the knowledge and skills here, they need to thinking about leading the team to deliver ECMO care in high quality when returning to their work location to establish an ECMO center in the future. (Participant 11, department head nurse)

Executive ability is quite needed. For example, if you have set the goal to complete quality management recently, you might go for it and achieve it on schedule. You cannot change it or do not finish it casually. (Participant 5, ECMO specialist)

ECMO nurses should summarize their experience with ECMO care regularly. Since the number of ECMO cases is still small and ECMO centers are not established in number at present, we need to accumulate some experience for colleagues in our center and other centers established in the future. (Participant 2, ECMO specialist)

Mastering organizing and coordinating is quite important during the ECMO care.

We are usually in charge of so many tasks during ECMO care. You need to make a list of priorities about your daily work schedule to needle patients' requirements and adjust flexibly. (Participant 7, ECMO specialist)

If a patient is going to take a rescue ECMO operation, you need to arrange the manpower quickly, including arranging the right person to the appropriate position, and clarifying the ability and responsibility of everyone. Besides, you have to manage not only the patient but also the whole ward. (Participant 5, ECMO specialist)

As an ECMO nurse, delivering humanistic care is significant for patients and their family.

We once treated a girl with fulminant myocarditis. We talked to her and calmed her down. Her leaders also paid more attention to her. The epidemic was not serious at that time, so we allowed her family to come in to visit her and encourage her. She got recovery finally. We did not use any sedative or analgesic drugs during the ECMO treatment. (Participant 9, department head nurse)

Multidisciplinary teamwork contributes to high quality.

After these materials, personnel and environment are prepared, we will participate in the cooperation during the puncture. Then, after establishing the ECMO, we will involve in the observation of the condition, which is our usual nursing work. Finally, we will assist the doctor in weaning from the ECMO and observe changes in the patient's condition after the weaning. We need to pay attention to the blood and fluid leaking from the puncture port because of extracting such a thick pipe. (Participant 19, ECMO specialist)

Security practice keeps ECMO nurses and patients safe.

Another thing that needs to emphasize is the writing of medical documents. Because even if you have performed well, you may fall into trouble due to the badly written documents when faced with a medical dispute. (Participant 5, ECMO specialist)

ECMO nurses need the innovative ability.

Maybe through some research or innovations, we could improve the ECMO nursing quality, such as dressing replacement methods and fixation innovations. We need the innovative ability to adapt the development of ECMO technology in the future. (Participant 4, department head nurse)

Assessing self‐performance regularly in ECMO care and timely seeking help are needed.

We sometimes encountered unexpected problems during the ECMO run. If you feel you can’t solve it, seek help promptly. (Participant 19, ECMO specialist)

ECMO nurses should not be satisfied with the status quo. Keeping learning and taking the initiative to stay on the frontier is needed. (Participant 14, ECMO physician)

#### 4.2.4 “Attitudes”‐related competencies

Attitudes were highly emphasized in ECMO nurses. There were nine subcompetencies of “attitudes.” The details are shown in Table 5.

Occupational loyalty is needed.

You have to love the job (ECMO care) in depth. As a result, you will try to manage the patient well and may try something innovative to help them, such as pain relief. Also, if you love the career, you will immerse yourself in it and not feel tired. (Participant 14, ECMO physician)

ECMO nurses are supposed to hold empathy:

The ICU was a closed environment. The family was not around. the patient could easily feel anxious. Maybe we would react in the same way. So we usually counseled her and tried to keep the ward quiet. (Participant 9, department head nurse)

Keeping self‐supervision is required.

Some colleagues behaved as unmotivated and lazy because they were just here for regular training. What we learned later was that they performed well in other wards. All the behaviors previously resulted from the unwillingness to work in ICU wards. (Participant 7, ECMO specialist)

The dedication was also needed.

We didn’t have no extra time to rest. I was supposed to go home and have a rest at 23 o’clock on schedule, but finally, we ended up completing the final rescued work at 4 o’clock on the second day. (Participant 5, ECMO specialist)

Psychological adjustment also plays an important role in smooth work.

ECMO treatment could be urgent. You need to adjust yourself and keep up with your physicians quickly. (Participant 20, ECMO specialist)

ECMO nurses are expected to keep careful and responsible in the daily delivery of ECMO care.

The ECMO machine alarmed suddenly, showing low flow, and stopped running finally. No one could find any reason. I happened to pass by and noticed that the pipe at the end of the bed was bent, so I immediately straightened the pipe. Finally, the machine ran smoothly and the patient got no impact. The nurse found it resulted from not checking on time after changing positions. (Participant 2, ECMO specialist)

ECMO nurses are also expected to be patient.

For ‘awake’ ECMO patients, we need to pay attention to their psychological needs, such as the desire for family, and physical needs, such as relieving pain. You may have to spend more time with them. (Participant 11, department head nurse)

Possessing physical fitness is a fundamental need for ECMO nurses.

At the moment completing resuscitation for this patient, another lung transplanted patient was also required rescue. Normally, we finish work at 23:00, but that day we ended up finishing at 4:00. It really demanded a strong physical fitness. (Participant 5, ECMO specialist)

## 5 DISCUSSION

### 5.1 Overview of ECMO nurses' competence

The aim of the study was to explore Chinese ECMO nurses' competencies in ECMO care. Guided by the Ground theory and the CIT, this study resulted in a competency framework containing knowledge, skills, behaviours and attitudes for ECMO nurses. Based on the framework of the Iceberg Model (Spencer & Spencer, 1993), we exhibited the competencies identified as a model, classifying those linked to “knowledge” and “skills” as the surface layer and “behaviours” and “attitudes” as the deeper layers of the model. This allows us to explain the structure of competencies related to ECMO care and visualize each layer in detail and its relationship with other layers. The “knowledge” was connected to these “skills.” Then ECMO nurses perform their behaviours to promote the ECMO nursing and guarantee safety based on the “knowledge” and “skills.” Last but not least, “attitudes” help perform in the best way we could. To our knowledge, this is the first published research investigating the competency of Chinese ECMO nurses.

The domain of “knowledge” encompasses three subcompetencies and 14 items while “skills” contains 11 subcompetencies and 28 items. As frontlines of ECMO care, patient–circuit interaction, addressing the patient's clinical needs, ensuring the safety of the ECMO circuit through constant surveillance, assessment and troubleshooting, and preventing and managing circuit emergencies are all their responsibilities (Daly et al., 2017). The subcompetencies contained in these domains in this study represent the related competencies that ECMO nurses should have. These subcompetencies are similar to those described in other studies for ECMO nurses, like interpersonal communication (Alsalemi et al., 2020; Alshammari et al., 2022), emergency handling skill (Alshammari et al., 2022; Boling et al., 2016; Patel et al., 2019), risk warning and prevention skill (Alshammari et al., 2022; OʼConnor & Smith, 2018) and so on. Due to the sensitivity and inherent risks of the operation, ECMO nurses who participated in this study recognized that competence in ECMO care benefits from the continuous training received before and experience in ECMO care, which help obtain adequate preparation and commitment. Besides, as it was reported that emergency handling skill has become a key competence for ECMO care (Alshammari et al., 2022; OʼConnor & Smith, 2018), this study gained the same result. Among the 40 episodes described in this study, 33 emphasized the emergency handling skill. In addition, it has been a consensus in the contemporary Chinese nursing community that evidence‐based practice is the cornerstone for improving the quality of clinical care (Chen et al., 2021; Xu et al., 2022). The acquisition of competencies in evidence‐based practice will be a key determinant for ECMO nurses to conduct quality care, which will ultimately contribute to the advancement of health care across the ECMO centre. The framework provides a supplemental guide to assess whether they have mastered solid knowledge and skills to ensure the quality of nursing care for ECMO patients.

In addition to requiring competencies involving knowledge and skills, participants perceived that “behaviours” and “attitudes” relevant competencies help form positive occupation cognition and lead to a positive development for ECMO multidisciplinary team. First, carrying out such a complex and challenging technique, leadership help organize the programmes and coordinate with others (Alsalemi et al., 2020). For example, trainees who prepare to implement ECMO programmes when returning to their communities need to learn how to form and lead an ECMO multidisciplinary team. Moreover, compared with models in some other countries (Alshammari et al., 2022; Daly et al., 2017), the ratio of nurses and ECMO patients is 1:1 in China, and ECMO nurses are under heavy pressure. When possessing a positive occupation in ECMO care, the nurses could get more deep power support from themselves, and be willing to undertake their work with great caution and attention, although this might impact their time schedules and general well‐being (Alshammari et al., 2022). Besides, psychological adjustment yielded in this study is also important for ECMO nurses due to the heavy pressure, which helps remind managers of concerning their psychological well‐being (Wellman, 2017). In addition, ECMO nurses were expected to stick to learning in this study to keep in touch with updated professional developments in ECMO management and try innovation, which was similar to previous research (Alshammari et al., 2022; Hackmann et al., 2017; OʼConnor & Smith, 2018). Recently, ECMO nurses in some other countries have proposed quantities of innovative ways and implemented them to overcome challenges met in ECMO care and comfort patients (Knisley et al., 2019; OʼConnor & Smith, 2018; Ohbe et al., 2018). Compared with them, research about ECMO nursing in China remains in experience summaries and case reports, which limits the development of ECMO care (Xu et al., 2021; Yang et al., 2020). Furthermore, as Chinese ECMO care mainly engages in the single caregiver model (Combes et al., 2014), ECMO nurses were expected to overcome difficulties generated by temporary insufficient experience and human resources in ECMO care to deliver daily care (Nie et al., 2020). The framework could help figure out the lack of other essential behaviours and attitudes during the ECMO care. Meanwhile, the leaders will thereby optimize training programmes and strengthen training for better performance.

### 5.2 Limitations

Nevertheless, this study has certain limitations. First, no representatives from ECMO patients or their family members were included. Patients and their family members' perspectives should be taken into account when developing a competency framework (Zhang et al., 2019). Second, we did not divide traits, values and self‐concepts in the deep layer. Traits, values and self‐concepts were included in “behaviours” and “attitudes,” but further investigation is necessary into these ability requirements. Ultimately, the framework should be clarified and simple to remember.

## 6 CONCLUSIONS

Using the semi‐structured interview based on the CIT, this study developed a comprehensive and detailed competency framework for Chinese ECMO nurses. It included four domains, encompassing 33 subcompetencies and 66 items. The four domains include knowledge, skills, behaviours and attitudes. It can be a reference for the training and assessment of ECMO nurses. Acceptance and implementation of the framework in clinical practice are needed. Future studies can serve the framework as an assessment to measure ECMO nurses' readiness and performance to highlight competences for improvement, thus promoting competence‐based development and supporting the delivery of best practices.

## AUTHOR CONTRIBUTIONS

All authors have approved the manuscript that is enclosed and also agreed to be accountable for all aspects of the work. The authors' contribution are as follows: HL, HC, CL, HX, HJ, LW: Study design and conception. HL, SX: Data acquisition. HL, SX: Data analysis and interpretation. HL, HC, CL, HX, HJ, LW: Manuscript writing. HC, LW: Supervision.

All authors have agreed on the final version and meet at least one of the following criteria [recommended by the ICMJE (http://www.icmje.org/recommendations/)]:

- Substantial contributions to conception and design, acquisition of data or analysis and interpretation of data;
- Drafting the article or revising it critically for important intellectual content.

## FUNDING INFORMATION

This work was supported by the 2022 Nursing Research Project of Chinese Medical Association Publishing House [grant numbers CMAPH‐NRP2021013].

## CONFLICT OF INTEREST

The authors declare no conflict of interest.

## ETHICAL APPROVAL

This study was approved by the Medical Ethics Commission of the First Affiliated Hospital of Guangzhou Medical University.The Ethical number is 2021‐122. All participants signed a consent form detailing the aim of the study, procedures, expected outcomes, risks and benefits to participate in this study and received a copy. Besides, all participants had the right to reject taking part.

## Supporting information


File S1

File S2
