# Supplementary material for: Developing a competency framework for extracorporeal membrane oxygenation nurses: A qualitative study
Source: Nurs Open. 2022 Dec 3;10(4):2449–63. doi: 10.1002/nop2.1502 (PMC10006586; doi:10.1002/nop2.1502)
Supplement: Supplementary file 1 — File S1 [file NOP2-10-2449-s001.doc]

Supplementary File 1 Consolidated criteria for reporting qualitative studies (COREQ): 32-item checklist

| No Item | Guide questions/description | Yes/No | Page in the manuscript |
| --- | --- | --- | --- |
| **Domain 1: Research team and reflexivity** |  |  |  |
| Personal Characteristics |  |  |  |
| 1. Interviewer/facilitator | Which author/s conducted the interview or focus group? | Yes. | Page 5. |
| 1. Credentials | What were the researcher’s credentials? e. g. PhD, MD | Yes. | Page 5. |
| 1. Occupation | What was their occupation at the time of the study? |  | Page 5. |
| 1. Gender | Was the researcher male or female? | Yes. | Page 5. |
| 1. Experience and training | What experience or training did the researcher have? | Yes. | Page 5. |
| Relationship with participants |  |  |  |
| 1. Relationship established | Was a relationship established prior to study commencement? | Yes. | Page 4. |
| 1. Participant knowledge of the interviewer | What did the participants know about the researcher? e.g. personal goals, reasons for doing the research | Yes. | Page 4-5. |
| 1. Interviewer characteristics | What characteristics were reported about the interviewer/facilitator? e.g. Bias, assumptions, reasons and interests in the research topic | Yes. | Page 6. |
| **Domain 2: study design** |  |  |  |
| Theoretical framework |  |  |  |
| 1. Methodological orientation and Theory | What methodological orientation was stated to underpin the study? e.g. grounded theory,  discourse analysis, ethnography, phenomenology, content analysis | Yes. | Page 3-6. |
| Participant selection |  |  |  |
| 1. Sampling | How were participants selected? e.g. purposive, convenience, consecutive, snowball | Yes. | Page 4-5. |
| 1. Method of approach | How were participants approached? e.g. face-to-face, telephone, mail, email | Yes. | Page 4-6. |
| 1. Sample size | How many participants were in the study? | Yes. | Page 7. |
| 1. Non-participation | How many people refused to participate or dropped out? Reasons? | Yes. | Page 7. |
| Setting |  |  |  |
| 1. Setting of data collection | Where was the data collected? e.g. home, clinic, workplace | Yes. | Page 4. |
| 1. Presence of non-participants | Was anyone else present besides the participants and researchers? | Yes. | Page 5. |
| 1. Description of sample | What are the important characteristics of the sample? e.g. demographic data, date | Yes. | Page7and Table1. |
| Data collection |  |  |  |
| 1. Interview guide | Were questions, prompts, guides provided by the authors? Was it pilot tested? | Yes. | Page 5. |
| 1. Repeat interviews | Were repeat interviews carried out? If yes, how many? | Yes. | Page 5-6. |
| 1. Audio/visual recording | Did the research use audio or visual recording to collect the data? | Yes. | Page 5-6. |
| 1. Field notes | Were field notes made during and/or after the interview or focus group? | Yes. | Page 5-6. |
| 1. Duration | What was the duration of the interviews or focus group? | Yes. | Page 5. |
| 1. Data saturation | Was data saturation discussed? | Yes. | Page 5. |
| 1. Transcripts returned | Were transcripts returned to participants for comment and/or correction? | Yes. | Page 5-6. |
| **Domain 3: analysis and findings** |  |  |  |
| Data analysis |  |  |  |
| 1. Number of data coders | How many data coders coded the data? | Yes. | Page 6. |
| 1. Description of the coding tree | Did authors provide a description of the coding tree? | Yes. | Page 6. |
| 1. Derivation of themes | Were themes identified in advance or derived from the data? | Yes. | Page 6-7. |
| 1. Software | What software, if applicable, was used to manage the data? | Yes. | Page 6. |
| 1. Participant checking | Did participants provide feedback on the findings? | Yes. | Page 6. |
| Reporting |  |  |  |
| 1. Quotations presented | Were participant quotations presented to illustrate the themes / findings? Was each quotation identified? e.g. participant number | Yes. | Page 8-15. |
| 1. Data and findings consistent | Was there consistency between the data presented and the findings? | Yes. | Page 8-15 and  Tables 2-5. |
| 1. Clarity of major themes | Were major themes clearly presented in the findings? | Yes. | Page 8-15. |
| 1. Clarity of minor themes | Is there a description of diverse cases or discussion of minor themes? | Yes. | Page 8-22. |
