# Supplementary material for: Developing a competency framework for extracorporeal membrane oxygenation nurses: A qualitative study
Source: Nurs Open. 2022 Dec 3;10(4):2449–63. doi: 10.1002/nop2.1502 (PMC10006586; doi:10.1002/nop2.1502)
Supplement: Supplementary file 2 — File S2 [file NOP2-10-2449-s002.docx]

Supplementary File 2 The semi-structured interview guideline

**1. The semi-structured interview guideline for ECMO specialist nurses included the following questions:**

(1) Could you please introduce your roles and responsibilities in ECMO care?

(2) What competencies do you think ECMO nurses need to be qualified? Could you please list the relevant knowledge and skills that you may need?

(3) What core competencies do you think for ECMO nurses require to deliver ECMO care?

(4) Could you share more than one impressive event you participated in during the ECMO care? Maybe it was a success or a regret. How did these events arise? Who was involved? How did you deal with it? What were your thoughts and feelings at the time? How did things turn out?

(5) What competencies do you think need to be improved and developed in the process of ECMO care? Do you have any personal career goals or direction?

**2. The semi-structured interview guidelines for physician experts were as followed:**

(1) In the process of ECMO treatment, what competencies do you think nurses should have in terms of materials preparation, pipes pre-flushing, cooperation of ECMO catheterization, management and monitoring of the process, and handling of ECMO-related emergencies? What knowledge does it involve?

(2) Among the nurses you cooperated with, were you satisfied with any nurses in managing ECMO patients? On a scale of 1-10, how would you rate it? Why were you satisfied? Could you share the related events? How did these events start? Who was involved? How did you deal with it? What were your thoughts and feelings at the time? How did things turn out?

(3) In view of the opportunities and challenges that critical care nurses and ECMO technology may face in the future, what competencies of ECMO nurses do you think can be developed or enhanced?

**3. The semi-structured interview guideline for department head nurses included the following questions:**

(1) Could you please introduce the roles and responsibilities of nurses in the delivery of ECMO care?

(2) What competencies do you think ECMO nurses need to be qualified? Could you please list the required relevant knowledge and skills?

(3) What core competencies do you think for ECMO nurses require to deliver ECMO care?

(4) Could you share more than one impressive event you participated in or other colleagues have shared with you during the ECMO care? Maybe it was a success or a regret. How did these events arise? Who was involved? How did you deal with it? What were your thoughts and feelings at the time? How did things turn out?

(5) What competencies do you think need to be improved and developed in the process of ECMO care? Do you have any personal career goals or direction for ECMO nurses?

**4. The semi-structured interview guideline for educators included the following questions:**

(1) What competencies do you need to consider when training ECMO nurses?

(2) What competencies do you think ECMO nurses need to be qualified? Could you please list the required relevant knowledge and skills?

(3) What core competencies do you think for ECMO nurses require to deliver ECMO care?

(4) What competencies do you think need to be improved and developed in the process of ECMO care? Do you have any personal career goals or direction for ECMO nurses?
